# Substrate vibrations mediate behavioral responses via femoral chordotonal organs in a cerambycid beetle

**DOI:** 10.1186/s40851-016-0053-4

**Published:** 2016-08-26

**Authors:** Takuma Takanashi, Midori Fukaya, Kiyoshi Nakamuta, Niels Skals, Hiroshi Nishino

**Affiliations:** 1 Department of Forest Entomology, Forestry and Forest Products Research Institute, Tsukuba, Japan; 2 Graduate School of Agricultural and Life Sciences, The University of Tokyo, Tokyo, Japan; 3 College of Bioresource Sciences, Nihon University, Fujisawa, Japan; 4 Department of Geosciences and Natural Resource Management, University of Copenhagen, Frederiksberg C, Denmark; 5 Research Institute for Electronic Science, Hokkaido University, Sapporo, Japan; 6 Present address: Department of Horticulture, Chiba University, Matsudo, Japan

**Keywords:** Behavior, Vibration, Sense organ, Coleoptera

## Abstract

**Background:**

Vibrational senses are vital for plant-dwelling animals because vibrations transmitted through plants allow them to detect approaching predators or conspecifics. Little is known, however, about how coleopteran insects detect vibrations.

**Results:**

We investigated vibrational responses of the Japanese pine sawyer beetle, *Monochamus alternatus*, and its putative sense organs. This beetle showed startle responses, stridulation, freezing, and walking in response to vibrations below 1 kHz, indicating that they are able to detect low-frequency vibrations. For the first time in a coleopteran species, we have identified the sense organ involved in the freezing behavior. The femoral chordotonal organ (FCO), located in the mid-femur, contained 60–70 sensory neurons and was distally attached to the proximal tibia via a cuticular apodeme. Beetles with operated FCOs did not freeze in response to low-frequency vibrations during walking, whereas intact beetles did. These results indicate that the FCO is responsible for detecting low-frequency vibrations and mediating the behavioral responses. We discuss the behavioral significance of vibrational responses and physiological functions of FCOs in *M. alternatus*.

**Conclusions:**

Our findings revealed that substrate vibrations mediate behavioral responses via femoral chordotonal organs in *M. alternatus*.

**Electronic supplementary material:**

The online version of this article (doi:10.1186/s40851-016-0053-4) contains supplementary material, which is available to authorized users.

## Background

Many animals are sensitive to substrate-borne vibrations. Vibration detection is an important sense that is used for intra- and interspecific interactions in diverse animal taxa [1, 2]. Specifically, vibrations transmitted through plants propagate well, allowing plant-dwelling animals to detect approaching conspecifics or predators without relying on other signals [3, 4]. Insects exhibit a range of behaviors in response to vibrations [1, 2]. A ‘startle response’ is a fast jerky movement with short latency elicited by vibrations; it is considered to be a preparatory behavior that enables locomotion to follow in a smooth behavioral sequence [5–7]. Vibrations may also elicit abrupt cessation of ongoing movements, such as freezing behavior or thanatosis (long-lasting freezing) [6–12]. The functional significance of vibration detection can be classified into: i) predator–prey interactions, including prey localization and antipredatory behavior, and ii) social interactions, including sexual signals, aggressive signals, and heterospecific signals [1, 2, 4, 13–17].

Although a number of studies have shown that coleopteran insects detect vibrations, and that they exhibit behavioral responses [11–16], the sense organs mediating such responses are largely unknown. An electrophysiological study showed that unidentified sensory neurons originating from the tibia and tarsus responded to low-frequency vibrations in Scarabaeidae, Carabidae, and Silphidae [18]. Studies of orthopteran insects showed that the primary organs sensitive to vibrations are internal mechanoreceptors, called ‘chordotonal organs’, located in the legs and other body appendages; these organs are also known to participate in the motor control of joints [8, 19–23].

The Japanese pine sawyer beetle, *Monochamus alternatus* (Coleoptera: Cerambycidae), is the vector of the pine wood nematode, which kills pine trees [24]. In this study, we investigated the behavioral responses of *M. alternatus* to vibrations and identified a chordotonal organ in the leg that detects vibrations transmitted through the tree.

## Methods

### Insects

Dead pine trees, *Pinus thunbergii* and *Pinus densiflora*, infested with larvae of *Monochamu*s *alternatus* were collected at the Forestry and Forest Products Research Institute and its Chiyoda Experimental Station in Ibaraki Prefecture, Japan in February to March during the 2006–2013 period, and were kept within a screen-caged house in natural conditions. In June through July, adults emerging from the dead pine logs were collected and kept separately in plastic cups (ca. 10 cm diam., 6 cm high) at 25 °C and 50–60 % relative humidity with a 16:8-h L:D cycle. A few twigs (ca. 10 cm long) of *P. thunbergii* and *P. densiflora* were provided as diet and replaced every 3–7 days. Male and female mature adults (>2 weeks old) were used. All behavioral experiments were performed at room temperature (23–26 °C) during the light-on period.

### Vibration stimuli and behavioral responses

Pulsed sine waves of 100 ms duration ranging from 25 Hz to 10 kHz were shaped by commercial software (0105; NF Corp., Yokohama, Japan). The duration included 5 ms rise and 5 ms fall times, irrespective of frequency. The vibration stimuli, continuously looped back by a function generator (WF1945; NF Corp.) at intervals of 900 ms, were applied to a beetle via a vibration exciter (type 4809; Brüel & Kjær, Nærum, Denmark) connected to a type 2718 power amplifier. Frequencies (Hz) and amplitudes (zero-to-peak accelerations, m/s^2^) of vibrations were measured by attaching a piezoelectric charge accelerometer (type 4371 or 4393; Brüel & Kjær) to the center of the steel plate on which the beetle was placed. The signals were amplified by a type 2692 conditioning amplifier and displayed on an oscilloscope (DS-8822P; Iwatsu Test Instruments Corp., Tokyo, Japan).

Behavioral responses of *M. alternatus* to vibration stimuli were observed under various conditions (Experiments 1–3), as follows.

#### Experiment 1

**Fig. 1:**
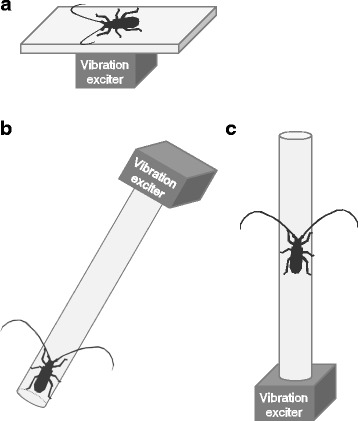
Setups for behavioral experiments. **a** An individual *Monochamus alternatus* beetle was allowed to move freely from a standstill on a steel plate attached to the vibration exciter (Experiment 1). **b** A naturally dried pine rod attached to the vibration exciter was tilted at ca. 70° from the horizontal. Freezing responses during walking or initiation of walking from a standstill were observed (Experiment 2). **c** The pine rod was attached vertically to the vibration exciter. Freezing responses were observed (Experiment 3)

An intact beetle was allowed to move freely on a flat steel plate (6 × 6 cm, 1 mm thick) attached to a vibration exciter placed on a desktop vibration isolator (UM-0405; Nippon Boushin Industry Co., Ltd, Numazu, Japan). Movements of the antennae and/or legs (startle response) and warning sound production using the prothorax and mesoscutum (stridulation) [16, 25] from a stationary position were categorized as behavioral responses to vibration stimuli in the inactive state. To determine the behavioral thresholds of these distinct responses, five male and five female beetles were used (Fig. 1a). The amplitude of the stimulus was gradually raised in 10-dB steps using the variable gain control of the power amplifier until a response appeared. The vibration-amplitude threshold was defined as the slightest acceleration necessary to evoke the response at a given frequency. When the response to vibration at a particular amplitude was clear, the response was counted. When the response to vibration was unclear, the same amplitude was applied again, with an inter-stimulus interval >1 min to avoid habituation. Six to nine different frequencies from 25 Hz to 10 kHz were presented to individuals in random order. After determining the threshold for a given frequency, subsequent thresholds for different stimulus frequencies were determined at intervals of >1 min. Immediately after each behavioral test, the acceleration on the center of the steel plate was measured as described above. No distinct differences in the thresholds were observed between the sexes; thus, the thresholds among different frequencies were analyzed in pooled individuals (*n* = 10) using the Kruskal–Wallis test, with multiple comparisons.

#### Experiment 2

Instead of the steel plate of Experiment 1, a rod of naturally dried pine trunk (3 cm diam., 30 cm long) attached to the vibration exciter with a screw (5 mm diam., 13 mm long) was hung from the ceiling of a soundproof box (90 × 90 × 70 cm) with a thick rope (Fig. 1b). The pine rod was tilted at ca. 70 ° from the horizontal. A beetle was gently transferred to the edge of the rod. Continuous waves of 100 Hz were applied to the beetle via the rod after it was allowed to rest or walk. Freezing responses during walking (i.e., cessation of walking), or initiation of walking from a stationary position were observed in intact beetles. Prior to the behavioral tests, vibration amplitudes as accelerations on the surface of the rod at 15 cm from the exciter were determined to be 0.03 m/s^2^. The numbers of freezing or walking behaviors in the presence of vibrations were compared with those in the absence of vibrations (control) using Fisher’s exact probability test.

#### Experiment 3

A naturally dried pine rod was attached vertically to the vibration exciter, which was placed on the desktop vibration isolator (Fig. 1c). Freezing responses during walking were observed in beetles with operated femoral chordotonal organs (FCOs), in sham-operated beetles (with femoral integument damaged with microscissors), and in intact beetles. The conditions used in this experiment were more suitable for observing the response than those in Experiment 2, because it allowed the beetles to walk up and down the rod. The FCO- and sham-operated beetles were allowed to recover for at least 1 day. As described in Experiment 1, the amplitude of stimuli at a set frequency was gradually raised from 0.01 to 7 m/s^2^ until a freezing response appeared. From an intact beetle, the threshold of the freezing response was determined as described in Experiment 1. After each behavioral test on intact beetles, the acceleration on the surface of the rod at 15 cm from the exciter was measured. Differences in the response at the same frequency were determined by Fisher’s exact probability test and Ryan’s multiple comparison test. The thresholds among different frequencies were tested by the Kruskal-Wallis test.

Although the exciter generated airborne sounds at frequencies above 500 Hz, the beetles did not exhibit any behavioral responses to sounds with similar frequencies and amplitudes broadcast from a speaker.

### Chordotonal organs

The FCOs and other chordotonal organs were stained by backfills from the main leg nerve (*n* = 12). The beetle was briefly anesthetized with carbon dioxide and then fixed ventral-side-up on a beeswax plate using insect pins. To stain peripheral nerves in the leg, the main leg nerve was cut at the terminus of the thoracic ganglion and its peripheral cut end placed into the tip of a tapered glass capillary tube filled with a 1 % micro-Ruby solution (MW = 3000; Invitrogen, Carlsbad, CA, USA). After fixation in 4 % paraformaldehyde solution for 6 h, specimens were dehydrated through an ethanol series, cleared in methyl salicylate, and viewed under a confocal microscope (LSM510; Zeiss, Jena, Germany). The stained chordotonal organs are shown in a false color (green). Optical sections (1.2 μm thick) were reconstructed two-dimensionally using commercial software (Amira ver. 3.1; FEI Visualization Sciences Group, Burlington, VT, USA) linked to the LSM510.

For FCO surgery, cell clusters (scoloparia) attached to the apodemes of all six femora of anesthetized beetles were carefully removed with microscissors immediately after opening a flap of the overlaying cuticle. The flaps were replaced to minimize damage to the surrounding muscles and tracheae. FCO-operated beetles were capable of walking although they exhibited deficits in the righting response (after turning them onto their backs) [26], which was slower than in intact beetles (6.8 s and 1.2 s, respectively; means of three measurements on two operated and two intact beetles) (Additional file 1: Video S1).

## Results

**Fig. 2:**
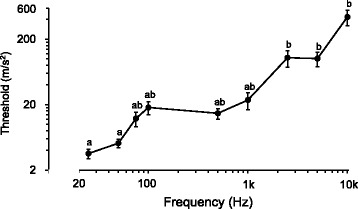
Behavioral thresholds to vibrations in *Monochamus alternatus*. Startle response (twitch movements) and stridulation from a standstill were categorized as behavioral responses to pulsed sine waves with different frequencies and amplitudes. Thresholds (mean ± SEM) with the same letters are not significantly different by the Kruskal-Wallis test with multiple comparisons (*p* > 0.05). For setup, see Fig. 1a

*Monochamus alternatus* exhibited startle responses (twitch movements) and/or stridulation when subjected to vibrations at different frequencies (Fig. 2). Vibrations frequently evoked startle responses, and occasionally stridulation with or without a startle response. The threshold progressively increased with frequency from 25 Hz to 1 kHz (Fig. 2), although the thresholds from 3.5 to 23.5 m/s^2^ were not significantly different. Higher amplitudes >100 m/s^2^ were needed to evoke the responses between 2.5 kHz and 10 kHz. Thus, the beetles were most sensitive to low frequencies (<1 kHz).

**Fig. 3:**
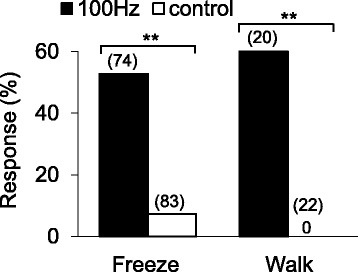
Proportions of *Monochamus alternatus* responding to vibrations at 100 Hz. The behavioral responses, freezing and walking, were significantly different from the controls in the absence of vibrations by Fisher’s exact probability test (**, *p* < 0.001). The numbers in brackets represent the total number of individuals. For setup, see Fig. 1b

Freezing or walking in response to vibrations was observed significantly more frequently than in the absence of vibrations in the controls (Fig. 3). The behavioral choice was state-dependent. When low-amplitude vibrations at 100 Hz were presented, 53 % of the beetles froze during walking, while 47 % did not freeze. Similarly, 60 % of the beetles initiated walking from a standstill in response to the vibrations, while 40 % did not. In contrast, in the absence of vibrations (controls), 7 % of the beetles froze and 0 % walked. The others showed no response. Startle responses to vibrations were rarely observed prior to walking and were never observed prior to freezing.

**Fig. 4:**
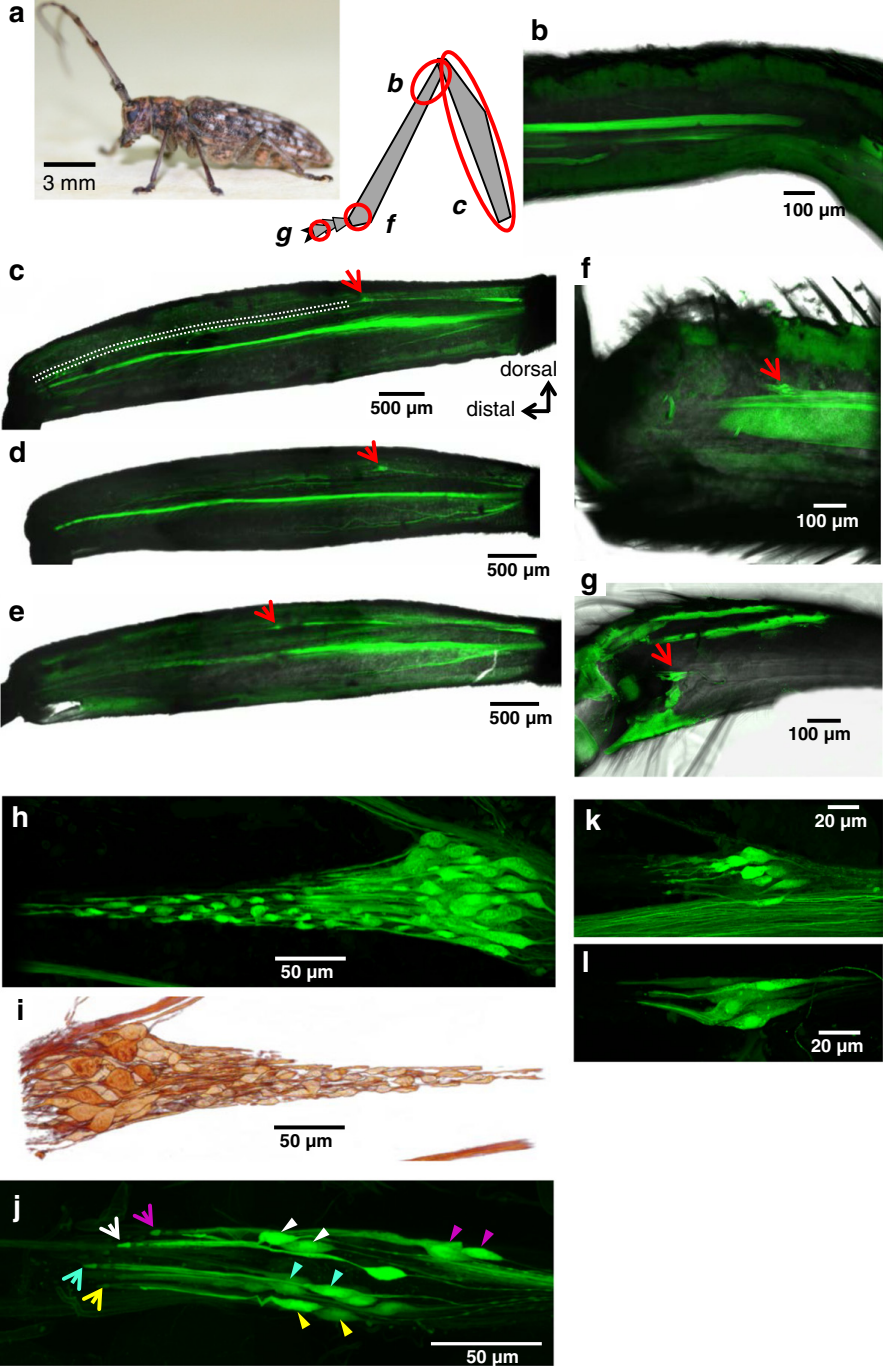
Leg chordotonal organs (COs) of *Monochamus alternatus*. **a** Photograph of female adult in resting posture, and diagram indicating the locations of COs in a femur (*c*), a tibia (*b*, *f*), and a tarsus (*g*). **b** Retrograde nerve staining of the prothoracic tibia showing absence of subgenual organ (*b* in **a**). **c**–**e** The FCO scoloparia (indicated by *red arrows*) in the prothoracic (**c**), mesothoracic (**d**), and metathoracic (**e**) legs. The cuticular apodeme is indicated by *dotted lines*. The FCO scoloparium in the metathoracic legs was located more distally compared with those in the pro- and mesothoracic legs. (**f**) The tibial CO in the distal region of the mesothoracic leg (*f* in **a**). **g** The tarsal CO in tarsal segment III of the metathoracic leg (*g* in **a**). **h** The prothoracic FCO scoloparium viewed posteriorly. **i** Three-dimensional reconstruction of the scoloparium **h**, viewed anteriorly. **j** High-power confocal stacks of the FCO in the mesothoracic leg. Pairs of cell bodies (*arrowheads*) inserted into common scolopale caps (*arrows*) are shown with different colors. **k** Magnified view of the tibial CO (**f**). **l** Magnified view of the tarsal CO (**g**)

Examination of backfills of the leg nerves at different proximo-distal levels revealed that the subgenual organ, a chordotonal organ specialized for vibration detection in orthopteran and heteropteran species [20, 27], is absent from all tibiae in *M. alternatus* (Fig. 4a*–*b), in agreement with a previous report on coleopteran species [18]. However, we identified the FCO as the largest chordotonal organ in each leg (Fig. 4a*–*g). The FCO shares general morphological characteristics with FCOs of other insects [20] but consists of a single scoloparium (cell cluster embedded in connective tissue) (Fig. 4h, i). The scoloparium of the FCOs in all legs was located in mid-femur (Fig. 4c*–*e), but the FCO scoloparium of the metathoracic leg was located more distally, resulting in a shorter cuticular apodeme than those of the pro- and mesothoracic legs. The FCOs contained about 60–70 sensory neurons (Fig. 4h, i) with no observable differences among the legs or between sexes. The FCO scoloparium was firmly attached to the anterior cuticle, and was distally connected to the proximal tibial joint via a ligament and the apodeme (Fig. 4c). A pair of neurons extended dendrites into a single scolopale cap (Fig. 4j), as previously reported for chordotonal sensilla of insect FCOs [20]. Compared with FCOs, the tibial and tarsal chordotonal organs were small and contained approximately 15 and six sensory neurons, respectively (Fig. 4k, l).

**Fig. 5:**
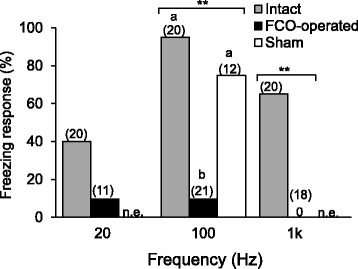
Proportions showing freezing responses to vibrations during walking in femoral-chordotonal-organ (FCO)-operated, sham-operated, and intact beetles. Significant differences in responses at the same frequency were detected with Fisher’s exact probability test (**, *p* < 0.001) and Ryan’s multiple comparison test for proportions at 100 Hz (different letters, *p* < 0.05). The numbers in brackets represent the total number of individuals. n.e.: not examined. For setup, see Fig. 1c

We compared freezing responses in FCO-operated, sham-operated, and intact beetles during walking (Fig. 5). When 100-Hz and 1-kHz vibrations were presented to intact beetles, a majority (95 and 65 %, respectively) showed freezing responses at various stimulus amplitudes. Similarly, 75 % of sham-operated beetles responded to 100 Hz. In contrast, only 9.5 and 0 % of FCO-operated beetles responded to 100 Hz and 1 kHz vibrations, respectively. Freezing responses to 100 Hz and 1 kHz differed significantly between the FCO-operated beetles and the other groups, but at 20 Hz the differences were not significant. The behavioral thresholds of intact beetles (mean ± SEM) were 0.41 ± 0.08 m/s^2^ at 20 Hz, 0.33 ± 0.06 m/s^2^ at 100 Hz, and 0.27 ± 0.06 m/s^2^ at l kHz (*n* = 8–18). There were no significant differences in thresholds among 20 Hz, 100 Hz, and l kHz.

## Discussion

*Monochamus alternatus* showed startle responses and stridulation from a standstill, when subjected to a broad range of vibrations below 1 kHz. In addition, the beetles froze or walked in response to vibrations at 100 Hz. Beetles with operated FCOs did not show freezing behavior, suggesting that the FCOs detect low-frequency vibrations and mediate this behavior. This finding is in accordance with a report that the cricket *Gryllus bimaculatus* with all FCOs operated tended not to exhibit long-lasting freezing behavior [8]. Freezing behavior mediated by excitation of sensory neurons in FCOs seems widespread across insects.

*Monochamus alternatus* showed different thresholds for the behavioral responses. Low-frequency vibrations of amplitudes 3.5–23.5 m/s^2^ induced the startle response and stridulation, whereas lower amplitudes of 0.3–0.4 m/s^2^ induced the freezing response. Similar differences in the stimuli needed to trigger the startle and freezing responses have been reported in *P. fortunei* [7]. Although the threshold was unclear, walking from a standstill was evoked by continuous vibrations with a low amplitude at 0.03 m/s^2^ in *M. alternatus*. Repeated exposures to vibrations above the threshold allow *M. alternatus* to walk, after initially showing the startle response.

What is the behavioral significance of the vibrational responses in *M. alternatus*? Some of the responses may be associated with anti-predator behavior. Approaching predators (e.g., birds [28]) cause low-frequency vibrations through a tree, which may elicit the startle and freezing responses mediated by the FCO of *M. alternatus*. For example, the cerambycid beetle *Hylotrupes bajulus* [16] and other insects [5, 9, 11] exhibit these responses, presumably as a defense against predators. Freezing and motionless insects are capable of hiding without emitting vibrational and/or other cues to predators [10, 11]. In addition, *M. alternatus* stridulates in response to vibrations. In cerambycids and other beetles, stridulation is regarded as a defensive, disturbance, or warning signal to potential predators [25, 29]. Startle and freezing responses may also serve for conspecific recognition in *M. alternatus*. Detection of approaching conspecifics by their vibrations could allow insects to prepare for subsequent behaviors, e.g., escaping or mating [30]. *P. fortunei* are able to detect vibrations from conspecifics landing and walking on the host leaf [7]. In addition to vibrations, contact sex pheromones and visual cues play important roles in conspecific recognition in cerambycids [31, 32]. Hence, vibrations may play an important role in both inter- and intraspecific interactions in concert with other sensory cues.

We identified for the first time the femoral chordotonal organ of a coleopteran species as a sensory organ detecting vibrations. In the absence of any specialized vibration detectors such as subgenual organs, the FCO, the largest leg chordotonal organ in *M. alternatus*, is suggested to play a pivotal role in the detection of low-frequency vibrations below 1 kHz. The FCO of *M. alternatus* possesses only a single scoloparium, which morphologically resembles the distal scoloparium in a locust [19, 20]. Considering that the distal scoloparia are sensitive to tibial movements and mediate reflexes in the leg muscles of a locust [19] and a stick insect [22], the FCOs of *M. alternatus* are likely to be bifunctional sensory organs that detect: i) small, fast small movements, e.g., accelerations through the tibia; and ii) large, slow movements, e.g., displacements of tibia. In fact, *M. alternatus* with operated FCOs took more time to right themselves (after turning them onto their backs) (Additional file 1: Video S1), an action that requires coordination of leg muscles. Possibly, pairs of neurons within a sensillum of the *M. alternatus* FCO have different physiological properties, as reported in the paired neurons of the antennal chordotonal organ in a cockroach [33]. Furthermore, the shorter apodeme of the metathoracic FCOs compared with the pro- and mesothoracic FCOs might be related to physiological properties (e.g., vibration detection, proprioception) in *M. alternatus*. Further studies are needed to determine the relationships between function and structure in *M. alternatus* FCOs.

## Conclusions

Our findings revealed that a cerambycid beetle showed behavioral responses, such as startle and freezing, when subjected to vibrations. For the first time, the internal mechanoreceptors, ‘chordotonal organs’, responsible detecting vibrations in a coleopteran species was identified. Micro-ablation of the femoral chordotonal organs in all legs completely abolished vibration-mediated freezing behavior. Freezing behavior may be associated with defense against predators.

## Additional file

Additional file 1: Video S1. Righting in FCO-operated and intact beetles. Beetles righted themselves after they were caused to fall upside down onto a sheet of paper. The movie was recorded with a Sony DCR-DVD201 Handycam at 30 frames per second. (MOV 3446 kb)

## Abbreviations

CO: Chordotonal organ
FCO: Femoral chordotonal organ
MW: Molecular weight
SEM: Standard error of mean
